# Insecticidal Potential of Defense Metabolites from *Ocimum kilimandscharicum* against *Helicoverpa armigera*

**DOI:** 10.1371/journal.pone.0104377

**Published:** 2014-08-06

**Authors:** Priyanka Singh, Ramesha H. Jayaramaiah, Priya Sarate, Hirekodathakallu V. Thulasiram, Mahesh J. Kulkarni, Ashok P. Giri

**Affiliations:** 1 Plant Molecular Biology Unit, Division of Biochemical Sciences, CSIR-National Chemical Laboratory, Pune, MS, India; 2 Chemical Biology Unit, Division of Organic Chemistry, CSIR-National Chemical Laboratory, Pune, MS, India; 3 CSIR-Institute of Genomics and Integrative Biology, New Delhi, India; 4 Proteomic Facility, Division of Biochemical Sciences, CSIR-National Chemical Laboratory, Pune, MS, India; Natural Resources Canada, Canada

## Abstract

Genus *Ocimum* contains a reservoir of diverse secondary metabolites, which are known for their defense and medicinal value. However, the defense-related metabolites from this genus have not been studied in depth. To gain deeper insight into inducible defense metabolites, we examined the overall biochemical and metabolic changes in *Ocimum kilimandscharicum* that occurred in response to the feeding of *Helicoverpa armigera* larvae. Metabolic analysis revealed that the primary and secondary metabolism of local and systemic tissues in *O. kilimandscharicum* was severely affected following larval infestation. Moreover, levels of specific secondary metabolites like camphor, limonene and β-caryophyllene (known to be involved in defense) significantly increased in leaves upon insect attack. Choice assays conducted by exposing *H. armigera* larvae on *O. kilimandscharicum* and tomato leaves, demonstrated that *O. kilimandscharicum* significantly deters larval feeding. Further, when larvae were fed on *O. kilimandscharicum* leaves, average body weight decreased and mortality of the larvae increased. Larvae fed on artificial diet supplemented with *O. kilimandscharicum* leaf extract, camphor, limonene and β-caryophyllene showed growth retardation, increased mortality rates and pupal deformities. Digestive enzymes of *H. armigera -* namely, amylase, protease and lipase- showed variable patterns after feeding on *O. kilimandscharicum,* which implies striving of the larvae to attain required nutrition for growth, development and metamorphosis. Evidently, selected metabolites from *O. kilimandscharicum* possess significant insecticidal activity.

## Introduction

Members of genus *Ocimum* have a unique blend of secondary metabolites which imparts them great medicinal properties as well as a peculiar flavor and taste [1]. Several members of *Ocimum* are known to possess antioxidant [2], antistress [3], anticancer [4], radiation protection [5], antifungal [6], antidiabetic [7], insecticidal [8] properties and other important bioactivities. *Ocimum* species abound in a diversity of secondary metabolites including terpenes, phenylpropanoids, phenolics etc., some of which may be involved in defensive roles. However, defense metabolites from these species have not been characterized, although, the insecticidal activity of the plant leaves against storage pests is reported [8]. Different species of *Ocimum* greatly differ in the composition of their secondary metabolites and may offer variable levels of resistance to specific insect pests. *Ocimum kilimandscharicum,* also known as camphor basil, is a relatively unexplored tropical plant species widely distributed in East Africa, India and Thailand. The species possesses a rich reservoir of secondary metabolites such as camphor, eucalyptol, limonene, geramacrene D and β-caryophyllene. These metabolites are reported to have insecticidal properties [9], [10], [11]. Thus, *O. kilimandscharicum* is an attractive system for studying potential insecticidal molecules.

Usually, insect infestation results in the reprogramming of both primary and secondary metabolism in plants. The roles of secondary metabolites in plant defense have been extensively studied and well documented [12]. However, the changes in primary metabolism that occur during infestation are equally important. Primary metabolites provide building blocks and energy molecules, all of which are required for defense pathways to function. Primary metabolites such as carbohydrates, proteins and lipids are also affected significantly during insect infestation. For example, the deposition of a plant polysaccharide callose is crucial for induced plant defense in rice and *Arabidopsis* [13], [14]. Similarly, large amount of callose deposition is evident in *O. basilicum* after phloem injury [15]. Plant proteins such as chitinases, enzyme inhibitors, and lectins have been well characterized and are known to aid in defense by repelling insects, inhibiting their feeding, or impairing their digestive or neural systems [16], [17]. Lipids or fatty acids (FAs) have direct and indirect roles in plant defense and function to provide biosynthetic precursors for cuticular components and jasmonic acid [18]. The fuel for producing secondary metabolites is derived from primary metabolites in the form of isopentenyl pyrophosphate (IPP), adenosine triphosphate (ATP), reduced nicotinamide adenine dinucleotide (NADH), etc.

Plant secondary metabolites are involved in several defense-related and other functions such as (i) prevention of herbivore and pathogen attack, (ii) attraction of pollinators and symbionts, [19] and (iii) plant-plant communication [20]. The diverse pool of secondary metabolites in genus *Ocimum* probably offers great resistance to biotic stresses. Unlike synthetic insecticides, plant-based bio-insecticides provide an organic, low-risk, environmentally friendly approach toward the management of insects in agriculture. Moreover, most of the terpenes and phenylpropanoids are ingredients of several medicinal formulations, and therefore their toxicity for mammals could be minimal [21]. The basil plant contains many useful secondary metabolites, which may prove to be important for the formulation of cost-effective bio-insecticides.

*Helicoverpa armigera* (Lepidoptera: Noctuidae) is a devastating insect pest that feeds on several economically important crop plants such as cotton, tomato, maize, chickpea, pigeon pea, etc. [22], [23]. *O. kilimandscharicum* is a non-host plant for *H. armigera*. Our earlier studies revealed the developmental and digestive flexibility in *H. armigera* fed on various diets [24], [25]. *H. armigera* regulates its enzyme levels to obtain better nourishment from its diet and avoid toxicity due to nutritional imbalance. Previous studies showed that ethyl acetate extracts of *O. canum* flowers and acetone extracts of *O. tenuiflorum* (previously *O. sanctum*) possess antifeedent and larvicidal characteristics, enabling them to act against *H. armigera* [26]. However, our knowledge of the interactions between *O. kilimandscharicum* and *H. armigera* is limited. The current study documents the changes in levels of primary and secondary metabolites in *O. kilimandscharicum* after *H. armigera* infestation. Furthermore, we have analyzed the responses of *H. armigera* larvae after feeding on *O. kilimandscharicum* metabolites.

## Materials and Methods

### Insect culture

*H. armigera* larvae were maintained on chickpea flour-based artificial diet under laboratory conditions (28±2°C and 75% relative humidity). The composition of the artificial diet was as follows: (A) 50 g chickpea flour, 5 g wheat germ, 12 g yeast extract, 3.5 g casein, 0.5 g sorbic acid, and 1 g methyl paraben in 150 mL distilled water, (B) 0.35 g choline chloride, 0.02 streptomycin sulphate, 2 g ascorbic acid, 0.15 g cholesterol, becadexamin multivitamin multi–mineral capsule (GlaxoSmithKline Pharmaceuticals Limited), 200 mg vitamin E, 1 mL formaldehyde, 0.3 g bavistin, 30 mL distilled water; and (C) 6.5 g agar in 180 mL distilled water. ‘A’ and ‘B’ were mixed together and molten agar ‘C’ was added to that mixture [27].

### Plant maintenance

*O. kilimandscharicum* and tomato plants (*var.* Abhinav) were grown in the greenhouse. The conditions in the greenhouse were as follows: temperature, 28 to 30°C; humidity, 35 to 40%; light conditions, 16 h light, 8 h dark.

### Feeding-choice assay

**Figure 1:**
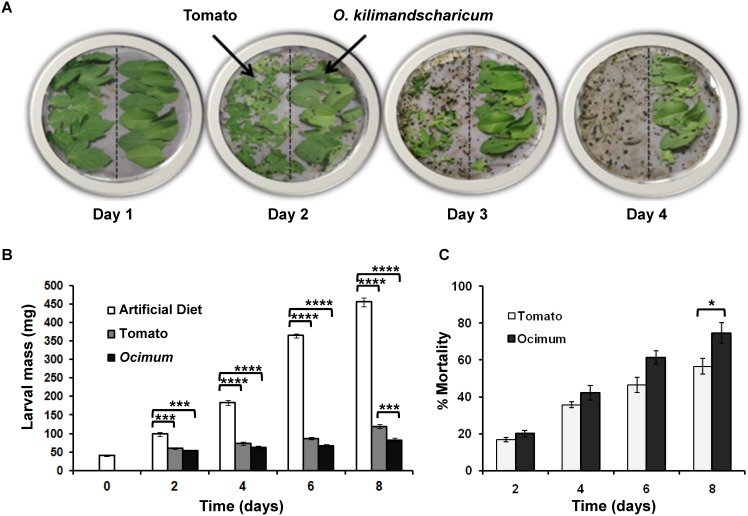
Performance of *H. armigera* feeding on tomato and *O. kilimandscharicum* leaves. **A.** Feeding assay showing feeding preference of *H. armigera* second-instar larvae for tomato over *O. kilimandscharicum* **B.** average larval mass and **C.** percentage mortality of larvae fed on artificial diet, tomato and *O. kilimandscharicum*. Two way ANOVA followed by Tukey's multiple comparisons test (Figure 1B) and Unpaired T test (Figure 1C) suggested significant difference between the data at *p*<0.0001 (indicated as ****), *p*<0.001 (indicated as ‘***’), *p*<0.05 (indicated as ‘*’).

One gram each of *O. kilimandscharicum* and tomato leaves were arranged in plastic Petri plates (15 cm diameter) opposite each other on moist filter paper. Second-instar *H. armigera* larvae were randomly transferred to the Petri plates (6 larvae/plate; n = 5). The amount of tissue remaining was noted each day at the same time for four days. The insects’ preference for a particular tissue type was proportional to the amount of tissue consumed. Greater consumption indicated greater preference in the choice assay (Fig. 1A).

### Growth and mortality data

*H. armigera* second- instar larvae were allowed to feed on artificial diet, tomato and *O. kilimandscharicum* plants individually. Five larvae per plant and 10 plants each of *O. kilimandscharicum* and tomato were infested with the larvae. Plants were covered with polythene bags, which were pierced with holes to allow respiration and maintained under the following greenhouse conditions: temperature, 28 to 30°C; humidity, 35 to 40%; light conditions, 16 h light, 8 h dark. For feeding on artificial diet, 50 larvae were maintained in vials (1 larvae/vial) containing equal amount of artificial diet. Percentage larval mortality and average increase in body mass were recorded every alternate day for 8 days.

### Biochemical and metabolite study

Second-instar *H. armigera* larvae were allowed to feed on *O. kilimandscharicum* plants (6 larvae/plant), 12 plants, for 6 days. Controls plants with no insects were also maintained. Control and test plants were covered with polythene bags, which were pierced with holes to allow respiration and maintained under the following greenhouse conditions: temperature, 28 to 30°C; humidity, 35–40%; light conditions, 16 h light, 8 h dark. Tissues were collected from the plants (local and systemic leaves, stem and roots) and larvae (whole larvae) after 12 h, 24 h, day 3 and day 6 and stored at −80°C till further use. The plant extracts for gas chromatography- mass spectrometry (GC-MS) were prepared using freshly harvested tissue that is described in further section.

### Estimation of carbohydrates, proteins, and lipids from plant tissues

The plant tissues (local and systemic leaves, stem and roots) collected at different time intervals (12 h, 24 h, day 3 and day 6) were analyzed for carbohydrates, proteins, and lipids. Total protein content was determined by the Kjeldahl method [28]. The phenol sulfuric acid method [29] was used to estimate total carbohydrate content using glucose as a standard. Total lipid content was measured using the sulpho–phospho–vanillin method [30]. All the assays were performed in duplicate and repeated thrice.

### *H. armigera* enzyme activity assays

Whole larval tissues (100 mg) were homogenized in 300 µL of 0.02 M sodium-phosphate buffer (pH 6.8) containing 10 mM NaCl for 2 h at 4°C. The homogenate was then centrifuged at 13,000×g for 30 min at 4°C. The supernatant was collected, stored at −20°C and used as crude enzyme source.

Total protease activity from *H. armigera* larvae fed on *O. kilimandscharicum* plants was assayed using azocasein as substrate [31]. Trypsin activity assays were performed as described by Tamhane et al. [32]. One protease unit was defined as the amount of enzyme in the assay that causes an increase in absorbance by one optical density under the given assay conditions. Amylase activity from the gut of *H. armigera* larvae was analyzed by the dinitrosalycylic acid method [33], as described by Kotkar et al. [24]. One amylase unit was defined as the amount of enzyme required to release 1 µM maltose/minute at 37°C under the given assay conditions. Lipase activity from gut homogenates was estimated using the p164 nitrophenyl palmitate assay [34]. One unit of lipase activity was defined as the amount of enzyme that causes an increase of one optical density under the given assay conditions. All the assays were performed in duplicate and repeated thrice.

### Extraction and analysis of metabolites

Plant tissue (1 g) (local and systemic leaves, stem and roots) was mixed in 10 mL dichloromethane (DCM) and kept for 18 to 24 h at 28°C. The extract was filtered and incubated for 2 h at −20°C to allow lipid precipitation. DCM extract was filtered again, concentrated under vacuum on a rotary evaporator and subjected to GC and GC-MS analysis.

GC analyses were carried out on an Agilent 7890A instrument equipped with a hydrogen flame ionization detector and an HP-5 capillary column (30 m×0.32 mm×0.25 µm, J and W Scientific). Nitrogen was used as the carrier gas at a flow rate of 1 mL/min. The column temperature was raised from 70°C to 110°C at 2°C min^−1^, then raised to 180°C at 3°Cmin^−1^ and finally to a temperature of 220°C with a 10°C min^−1^ rise; here it was held for 2 min. Injector and detector temperatures were 230°C and 250°C, respectively. GC-MS was performed on a HP 5975C mass selective detector interfaced with a HP 7890A gas chromatograph. GC-MS analyses were performed under similar conditions using an HP-5 MS capillary column (30 m×0.32 mm×0.25 µm, J and W Scientific) with helium as the carrier gas. Compounds were identified by comparing the retention time and mass fragmentation pattern of the standards of major constituents and also by comparing acquired mass spectra and retention indices with NIST/NBS and the Wiley mass spectral library (software version 2.0, Dec. 2005).

### *H. armigera* larvae fed on specific compounds

*O. kilimandscharicum* leaf extract and candidate compounds (camphor, limonene, **β**-caryophyllene, procured from Sigma, St. Louis, MO, USA) were dissolved in 30% dimethyl sulfoxide (DMSO) and incorporated in artificial diet at final concentration of 10, 100 and 1000 ppm. Diet prepared with equivalent amount of 30% DMSO was used as control. Larvae (20 per diet) were maintained individually in vials. Percentage larval mortality and average larval body mass were recorded every alternate day up to pupation. Pupal deformities were also recorded.

### Statistical analysis

Significant differences between diet treatments were determined using one-way ANOVA or two-way ANOVA followed by Tukey's multiple comparison and mentioned in respective figure legends. Unpaired T test was used to compare data from two treatments i. e. tomato and *O. kilimandscharicum* in respective analysis and to compare metabolic changes in local and systemic tissue, and detailed information provided in respective figure legends. One way ANOVA and Unpaired t-test data was considered to be significantly different within the treatments if the F-value obtained was higher than the critical F-value at *p*<0.001, *p*<0.01, *p*<0.05. Small letters are used to indicate statistically different groups of treatments. NS represents non-significant difference within the treatments and/or in the respective day.

## Results and Discussion

### *O. kilimandscharicum* defense compounds deter larvae from feeding, adversely affecting their growth and development

Feeding- choice assays showed that *H. armigera* larvae consumed significantly less *O. kilimandscharicum* leaf tissue than tomato (**Fig. 1A**). By the end of the fourth day, larvae had consumed all the tomato leaves and showed lower preference for *O. kilimandscharicum* leaves. Such resistance to feeding on *O. kilimandscharicum* leaves by *H. armigera* larvae clearly indicates the presence of defense compounds, which strongly deter larval feeding. It was also observed that larvae fed on *O. kilimandscharicum* plants showed significant growth impairment as well as an increase in mortality. These results are statistically supported by two way ANOVA followed by Tukey’s multiple comparison test and Unpaired T test respectively (**Fig. 1B and 1C**). Results of the two way ANOVA show a statistically significant interaction between treatments and larval mass at various days for a total variance of 28.27% at *p*<0.0001 (**Table 1** **and** **Fig. 1B**). The average body mass of larvae fed on *O. kilimandscharicum* was consistently lower on day 2, 4 and 6 than larvae fed on tomato but on day 8 it was significantly lower (at *p*<0.001 ‘***’). As expected, control (artificial) diet fed larvae showed significantly higher body mass (at *p*<0.0001 ‘****’) as compared to *O. kilimandscharicum* and tomato fed larvae on all days (**Fig. 1B**). No significant difference was observed in the mortality of *O. kilimandscharicum* and tomato fed larvae on day 2, 4 and 6. Although tomato is a host plant for *H. armigera*, it is known that larvae prefer to feed on the tomato fruit. Mortality of larvae fed on tomato leaves may be attributed to the presence of defense proteinaceous molecules like proteinase inhibitors or secondary metabolites. Results clearly indicate that the insects were unable to counteract the action of potential defense metabolites (**Fig. 1C**). Overall growth in *H. armigera* larvae fed on *O. kilimandscharicum* was slowed, possibly owing to the presence of defense metabolites.

**Table 1:** Two way analysis of variance for performance of *H. armigera* on various days feeding on tomato and *O. kilimandscharicum* leaves.

ANOVA table	SS	DF	MS	F (DFn, DFd)	P value
Interaction	195388	8	24424	F (8, 24) = 113.6	P<0.0001
Time	201904	4	50476	F (4, 24) = 234.8	P<0.0001
Diets (AD, TO, OC)	290117	2	145058	F (2, 6) = 2400	P<0.0001
Subjects (matching)	362.7	6	60.45	F (6, 24) = 0.2812	P = 0.9402
Residual	5160	24	215		
Total	692931	44			

DF = Degrees of freedom, SS = Sum of squares, MS = Mean square, n = numerator, d = denominator, p = probability of significance, α = 0.05.

### Changes in protein, carbohydrate and lipid content in *O. kilimandscharicum* upon insect attack

Different parts of *O. kilimandscharicum* plant were analyzed after larval infestation over a period of six days to estimate the changes in total carbohydrate, protein and lipid content. Two way ANOVA followed by Tukey’s multiple comparisons test showed significant interaction for the total varience of 13.32%, 27.23% and 31.11% at *p*<0.0001 between the up and down regulation of primary metabolites (i.e. protein, carbohydrate and lipase respectively) in different tissue (leaf, stem and root respectively) and the days of infestation (**Table 2** **and Fig. S1**). Protein content in *O. kilimandscharicum* leaves increased significantly during 12 and 24 h following insect infestation (**Fig. S1A**). Moreover, protein content increased in systemic leaves compared to in local leaves (**Fig. S2A**). However, the protein content decreased progressively as time increased. A similar trend was observed in stem and root tissues. The early increase in protein content might be a part of induced plant defense. A similar trend in lipid content was observed in all tissues. The carbohydrate content in *O. kilimandscharicum* plants increased two-fold in the first 24 h following infestation as compared to the carbohydrate content in uninfested plants (**Fig. S1B**), and subsequently remained the same pattern. The sudden increase in carbohydrate content confirms previous reports, which state that sugars play an important role in induced- defense by acting as important signaling molecules [35], [36].

**Table 2:** Two way analysis of variance for macromolecular content of *O. kilimandscharicum* leaves, stem and root on various days of *H. armigera* infestation.

ANOVA table	SS	DF	MS	F (DFn, DFd)	P value
**Protein**					
Interaction	485.4	8	60.68	F (8, 12) = 106.0	P<0.0001
Time	1820	4	455.1	F (4, 12) = 795.2	P<0.0001
Tissue (Leaf, stem, root)	1325	2	662.4	F (2, 3) = 311.0	P = 0.0003
Subjects (matching)	6.39	3	2.13	F (3, 12) = 3.722	P = 0.0422
Residual	6.867	12	0.5723		
Total	3644	29			
**Carbohydrate**					
Interaction	75461	8	9433	F (8, 36) = 199.3	P<0.0001
Time	86421	4	21605	F (4, 36) = 456.6	P<0.0001
Tissue (Leaf, stem, root)	113466	2	56733	F (2, 9) = 4273	P<0.0001
Subjects (matching)	119.5	9	13.28	F (9, 36) = 0.2806	P = 0.9759
Residual	1704	36	47.32		
Total	277170	59			
**Lipid**					
Interaction	446.9	8	55.86	F (8, 36) = 40.37	P<0.0001
Time	794.9	4	198.7	F (4, 36) = 143.6	P<0.0001
Tissue (Leaf, stem, root)	137.2	2	68.58	F (2, 9) = 78.34	P<0.0001
Subjects (matching)	7.878	9	0.8753	F (9, 36) = 0.6325	P = 0.7617
Residual	49.82	36	1.384		
Total	1437	59			

DF = Degrees of freedom, SS = Sum of squares, MS = Mean square, n = numerator, d = denominator, p = probability of significance, α = 0.05.

However, with a decrease in aerial tissues, the carbohydrates might relocate to the roots; this could explain the significant increase in the carbohydrate content of the root tissue on the sixth day (**Fig. S1B**). Schwachtje et al. [37] reported that *Nicotiana attenuata* plants divert their resources to less vulnerable tissues within the plant such as roots as a part of their defense strategy. We observed significantly more carbohydrates accumulation in systemic leaf tissue than in local tissue (**Fig. S2B**). This could be the plant’s way to protect its non-damaged plant parts by mobilizing resources and defense compounds. It was previously demonstrated that after a plant is injured or wounded by herbivore attack, local tissues signal systemic tissues to increase the plant's defense activity [38], [39]. From these observations, it can be hypothesized that *O. kilimandscharicum* adopts a carbohydrate-mediated defense strategy to combat insect infestation, a strategy that exists at the level of primary metabolism. The lipid content of *O. kilimandscharicum* leaves increased significantly during 12 and 24 h following infestation and then gradually declined (**Fig. S1C**). Furthermore, insect infestation was found to be responsible for the accumulation more lipids in systemic leaves as compared to local leaves (**Fig. S2C**). According to earlier reports, both 16- and 18-carbon fatty acids are known to modulate basal, effector-triggered and systemic immunity in plants. A sudden increase of lipid content in leaves of *O. kilimandscharicum* indicated the onset of secondary metabolite formation as a part of plant defense. Although basil is rich in secondary metabolites, no such details are available for the fatty-acid derived plant defense in *O. kilimandscharicum*.

### *H. armigera* regulates its digestive enzymes after feeding on *O. kilimandscharicum*

**Figure 2:**
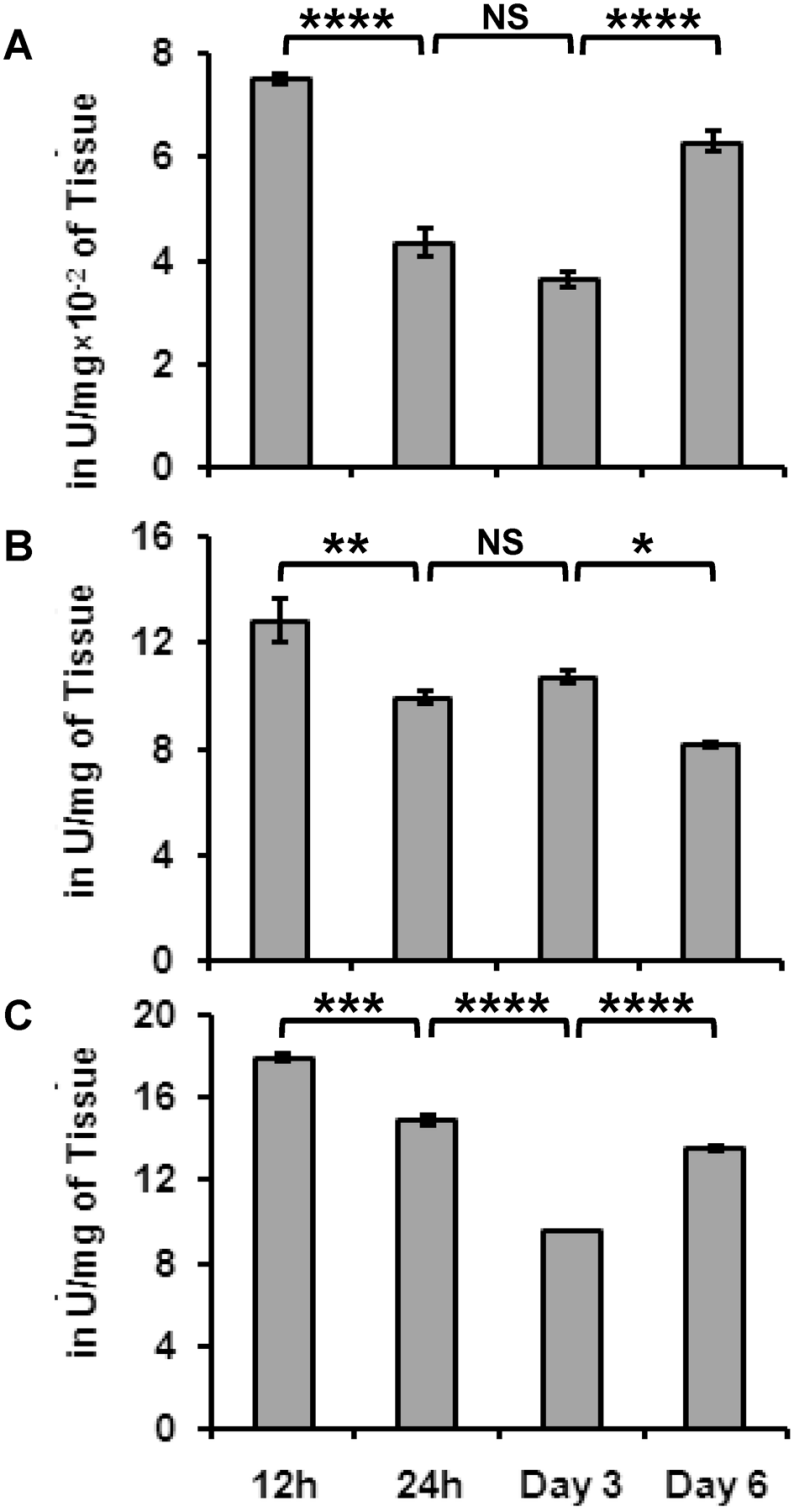
Digestive enzymes of *H. armigera* larvae fed on *O. kilimandscharicum* leaves. Changes in the levels of **A.** protease **B.** amylase **C.** lipase activity of *H. armigera* second-instar larvae fed on *O. kilimandscharicum* plants at 12 h, 24 h, day 3 and day 6. One way ANOVA followed by Tukey's multiple comparisons test suggested significant difference between the data at. *p*<0.001 (indicated as ‘***’), *p*<0.01 (indicated as ‘**’), *p*<0.05 (indicated as ‘*’). Results are an average of three independent experiments conducted in duplicate. Error bars represent Mean ± SD.

One way ANOVA followed by Tukey's multiple comparisons test suggested significant difference between the expression of protease, amylase and lipase in insect gut on 12 h, 24 h, day 3 and 6. The total protease activity of larvae fed on *O. kilimandscharicum* was measured at various time intervals. Initially, protease activity was found to decrease beginning at 12 h after feeding and continuing to the third day of feeding (**Fig. 2A**); however, protease activity increased dramatically on the sixth day of feeding. The initial decrease in protease activity can be attributed to the increased expression of inhibitory proteins in *O. kilimandscharicum*. The digestive track of insect is enriched with cocktail of proteases to utilize plant proteins and obtain amino acids for nutrition from plants. Moreover, plant defensive proteins also play a significant role in modulating the expression of insect proteases. Therefore, the higher protease activity observed on the sixth day after feeding might be indicative of the attempts of *H. armigera* larvae to obtain more nutrition from the ingested plant food. The plants produced antifeedent and antinutritive compounds that might be responsible for significant differences in amylase, protease and lipase activities in *H. armigera* larvae fed on *O. kilimandscharicum*. Amylase activity was examined during all the feeding assays (**Fig. 2B**). The amylase activity found in larvae correlated with the carbohydrate content of *O. kilimandscharicum*, which remained significantly high. Possibly, *H. armigera* maintains its amylase activity to better utilize the higher amount of carbohydrates from *O. kilimandscharicum* [24], [25].

A gradual decline in lipase activity was observed in larvae fed on *O. kilimandscharicum* from 12 h after feeding to the third day (**Fig. 2C**). Lipase activity measured in *H. armigera* larvae fed on *O. kilimandscharicum* was correlated with lipid content in the plant. The current study is consistent with our previous findings which revealed the differential expression of proteases, amylases and lipases in *H. armigera* in response to different diets [25].

### Compounds associated with secondary metabolism are central to *O. kilimandscharicum* defense

**Figure 3:**
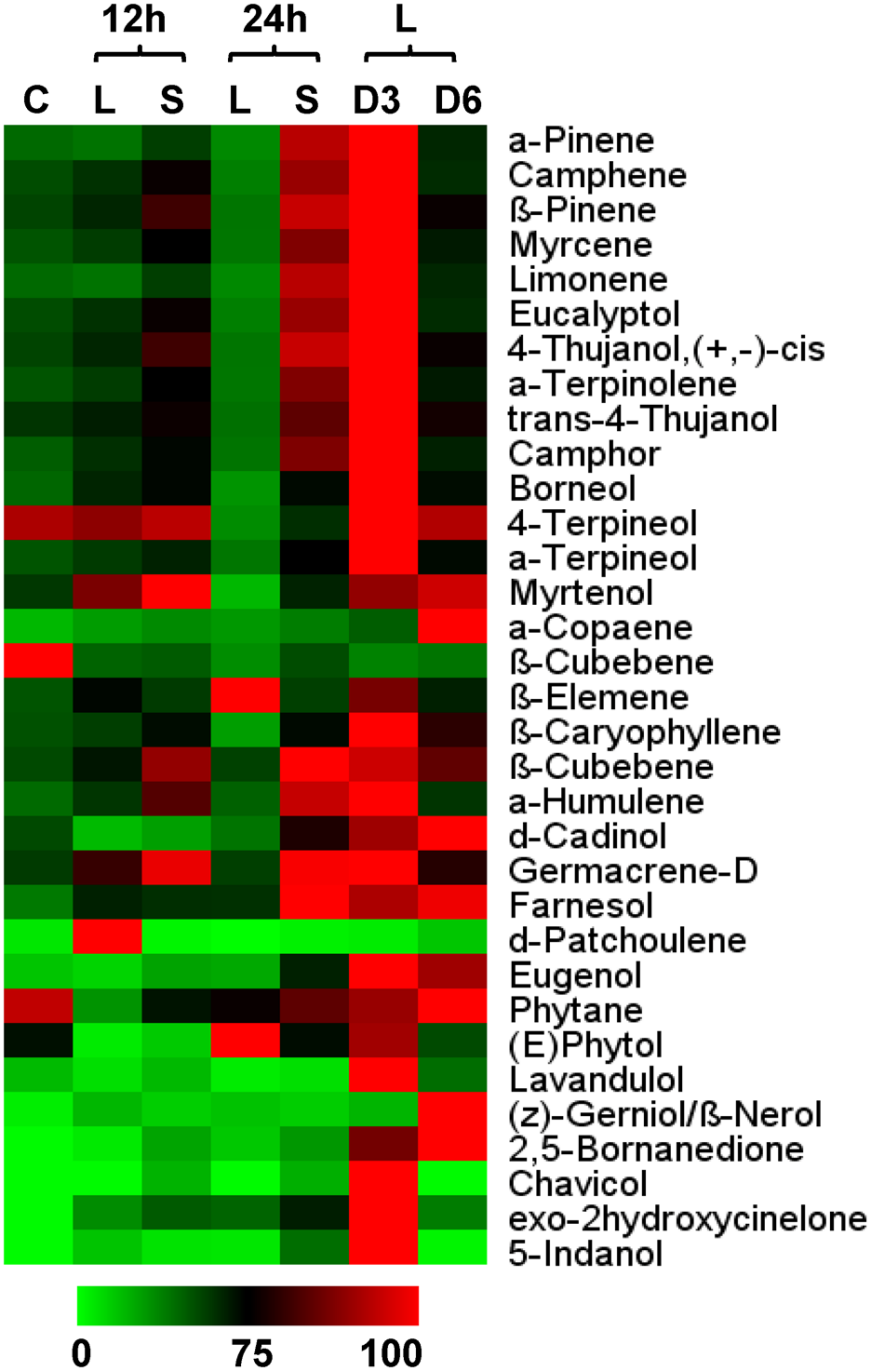
Metabolic changes in leaves of *O. kilimandscharicum* following *H. armigera* infestation. Heat map representing relative expression of a sub-set of volatiles elicited in leaf tissue during *O. kilimandscharicum*-*H. armigera* interaction; comparison between metabolite profiles of local (L) and systemic (S) leaf tissue in *O. kilimandscharicum*, 12 h and 24 h after feeding by *H. armigera*, and also on days 3 (D3) and 6 (D6), compared to control (C) plants.

Consistently increasing accumulations of monoterpenes, sesquiterpenes, phenylpropanoids and hydrocarbons were evident in the leaves of *O. kilimandscharicum* from 12 h to day 3 after insect infestation (**Fig. 3**). The maximum defense response was elicited on the third day, when levels of all the metabolites were higher. However, the accumulation of metabolites decreased progressively towards day 6. When a few leaves are left, plants mobilize their resources in the direction of their stems and roots. However, metabolite accumulation in systemic leaf tissues was higher than in local tissues in 12 and 24 h after insect attack (**Fig. 3**).

**Figure 4:**
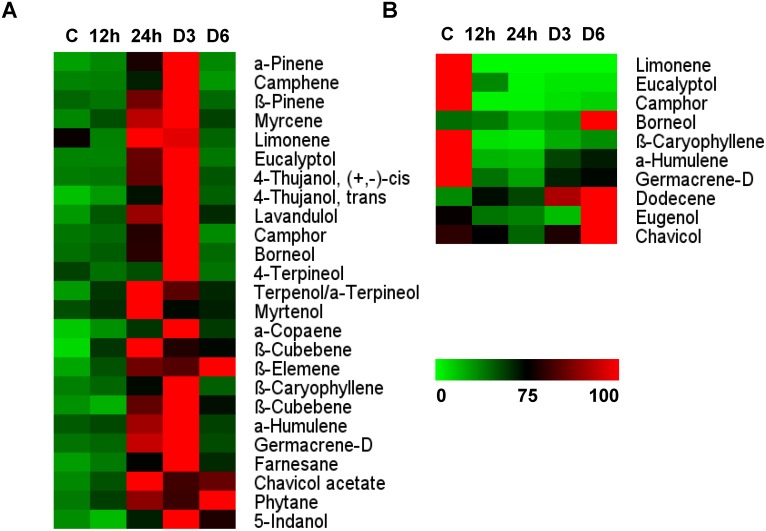
Metabolic changes in stems and roots of *O. kilimandscharicum* following *H. armigera* infestation. Heat map representing relative expression of a sub-set of volatiles elicited in **A.** stems and **B.** roots during *O. kilimandscharicum*- *H. armigera* interaction at 12 h, 24 h, and on days 3 (D3) and 6 (D6) as compared to control (C) plants.

Changes in the levels of metabolites observed in the stem follow a pattern similar to that in leaves (**Fig. 4A**). The defense response was high on the third day, so the plant mobilized all its reserves in the roots, and hence fewer metabolites were detected in the stem on the sixth day. Generally, the stem contains fewer metabolites than leaves. Our results suggest that the stem seems to play the role of translocator. The stem transports metabolites from roots to leaves during the initial defense response and channels metabolite reserves to the roots during later stages of infestation when the aerial tissues are consumed.

Few compounds were detected in roots, such as camphor, eucalyptol, limonene, eugenol, geramacrene D and humulene. Levels of these metabolites decreased initially (12 h post-infestation) and were minimal at 24 h (**Fig. 4B**). This probably happened because defense metabolites present in the roots were mobilized to the leaves, which need to be protected from the insect feeding and damage. However, the metabolite concentration in roots gradually increased between days 3 and 6. The metabolic pool might be channeled back to the roots if the aerial parts are destroyed.

### *O. kilimandscharicum* metabolites cause severe pupal deformities in *H. armigera*

**Figure 5:**
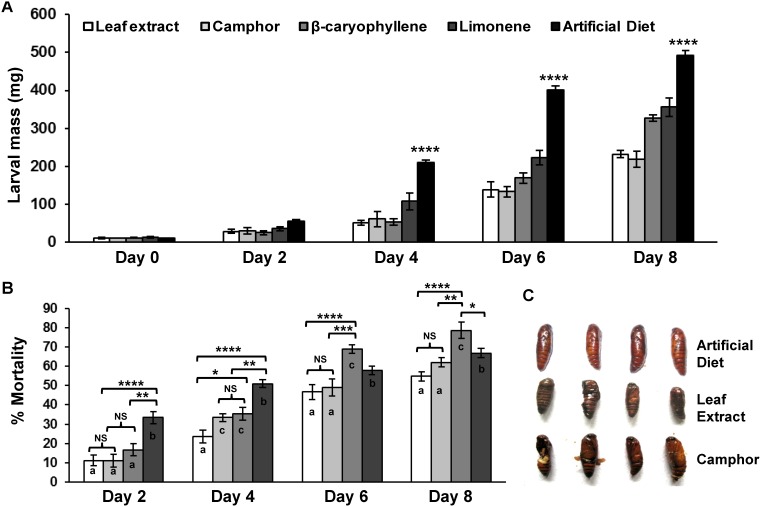
Antibiosis to *H. armigera* following exposure to *O. kilimandscharicum* leaf extract and selected compounds. **A.** Average larval mass and **B.** percentage of mortality of *H. armigera* second-instar larvae fed on artificial diet supplemented with *O. kilimandscharicum* leaf extract, camphor, limonene, β-caryophyllene at 100 ppm (day 0 to 8) and **C.** pupal deformities and death for larvae fed on diet supplemented with *O. kilimandscharicum* extract and camphor. Two way ANOVA followed by Tukey's multiple comparisons test between the treatment and larval mass (A) at different time points suggested significant interaction at *p*<0.0001. Significant difference in data at *p*<0.0001 (indicated by ****), at *p*<0.001 (indicated by ***), at *p*<0.01 (indicated by **), at *p*<0.05 (indicated by *). Small letters in (B) represents results for Tukey’s post hoc test. Similar alphabets in column represent group of diets showing non-significant (NS) difference in mortality while different alphabets represents diets exhibiting statistically different mortality in that particular day. NS represents non-significant difference in mortality.

To measure the insecticidal performance of individual defense metabolites from *O. kilimandscharicum*, feeding assays were carried out with *H. armigera* second instar larvae. Results of two way ANOVA show a statistically significant interaction for the total variance of 9.95% at *p*<0.0001 between the days of infestation and growth of larvae fed on leaf extract, camphor, limonene, β-caryophyllene, artificial diet (**Fig. 5A** **,** **Table 3**) and also for mortality (for the total variance of 5.96% at *p* = 0.0009) (**Fig. 5B** **,** **Table 3**). Growth was retarded in all larvae fed on the diet supplemented with selected metabolites on all days. Artificial diet fed larvae showed significantly more larval mass as compared to larvae fed on the other diets at day 4, 6, and 8 (at *p*<0.0001) (**Fig. 5A**). Larvae fed on the selected metabolites exhibited different percentage mortality. Larvae fed on limonene-based diet showed significantly more mortality (forming separate group ‘b’) as compared to other three diets on day 2 and 4 (at *p*<0.01, *p*<0.0001) whereas β-caryophyllene showed significantly more mortality compared to other diets (forming separate group ‘c’) on day 6 and 8 (at *p*<0.05, *p*<0.01, *p*<0.0001). Significantly less growth and high mortality in four diets (leaf extract, camphor, limonene, β-caryophyllene) fed larvae indicates gradual effect of these compounds on insect survival (**Fig. 5B**). Additionally, pupal deformities were evident in the insects fed on camphor and *O. kilimandscharicum* leaf extract (**Fig. 5C**). Our results clearly show that these metabolites can directly affect insect growth, survival and pupation, and hence can be used as potent insecticides.

**Table 3:** Two way analysis of variance for growth inhibition and percentage mortality of *H. armigera* upon exposure to *O. kilimandscharicum* leaf extract and selected metabolites on various days.

ANOVA table	SS	DF	MS	F (DFn, DFd)	P value
**Growth inhibition**					
Interaction	140388	16	8774	F (16, 40) = 32.79	P<0.0001
Time	1.04E+06	4	259175	F (4, 40) = 968.6	P<0.0001
Treatments	210262	4	52566	F (4, 10) = 41.55	P<0.0001
Subjects (matching)	12651	10	1265	F (10, 40) = 4.728	P = 0.0002
Residual	10704	40	267.6		
Total	1.41E+06	74			
**% Mortality**					
Interaction	1246	9	138.4	F (9, 24) = 4.838	P = 0.0009
Time	16021	3	5340	F (3, 24) = 186.6	P<0.0001
Treatments	2732	3	910.5	F (3, 8) = 33.80	P<0.0001
Subjects (matching)	215.5	8	26.94	F (8, 24) = 0.9415	P = 0.5019
Residual	686.7	24	28.61		
Total	20900	47			

DF = Degrees of freedom, SS = Sum of squares, MS = Mean square, n = numerator, d = denominator, p = probability of significance, α = 0.05.

## Conclusion

*O. kilimandscharicum* elicited a strong defense response to counteract *H. armigera* larval infestation. The defense-associated metabolites such as monoterpenes, sesquiterpenes and phenylpropanoids were upregulated. The growth and development of *H. armigera* larvae was significantly retarded when they fed on *O. kilimandscharicum* leaves as compared to tomato leaves. Initially, primary metabolism in *O. kilimandscharicum* was drastically affected by insect infestation as was evident from the increased concentration of carbohydrates. Moreover, metabolites such as camphor, β-caryophyllene, terpinolene and limonene increased greatly during infestation. This increase might be attributed to the plant’s strong insecticidal properties [40], [41]. Importantly, selected compounds from *O. kilimandscharicum* leaves were also able to retard larval growth and induce pupal deformities in *H. armigera*. We conclude that defense metabolites from *O. kilimandscharicum* possess strong insecticidal activity even at lower concentrations revealed by present study and corroborated by earlier reports [40], [41].

## Supporting Information

Figure S1 **Protein, carbohydrate and lipid content of** ***O. kilimandscharicum*** **leaves** ***following H. armigera*** **feeding.** Changes in the levels of **A.** total proteins **B.** total carbohydrates **C.** total lipids in leaves, stems and roots of tomato and *O. kilimandscharicum* at 12 h, 24 h, day 3, and day 6 post-infestation by *H. armigera* second-instar larvae. Two way ANOVA followed by Tukey's multiple comparisons test suggested significant difference between the data at. *p*<0.001 (indicated as ‘***’), *p*<0.01 (indicated as ‘**’), *p*<0.05 (indicated as ‘*’). One color represents data from respective day. NS represents group with non-significant difference in that particular day. Error bars represent Mean ± SD of 4 independent sets of tissue samples.
(TIF)

Figure S2 **Protein, carbohydrate and lipid content of** ***O. kilimandscharicum*** **leaves following** ***H. armigera*** **feeding.** Changes in the levels of **A.** total proteins **B.** total carbohydrates **C.** total lipids in local (L) versus systemic (S) leaf tissue in *O. kilimandscharicum* at 12 and 24 h post-infestation by *H. armigera* second-instar larvae. Unpaired t test suggested significant difference between the local and systemic tissue analysis data at. *p*<0.001 (indicated as ‘***’), *p*<0.05 (indicated as ‘*’). Error bars represent Mean ± SD of 4 independent sets of tissue samples.
(TIF)
