# Supplementary figures and images for: Insecticidal Potential of Defense Metabolites from Ocimum kilimandscharicum against Helicoverpa armigera
Source: PLoS One. 2014 Aug 6;9(8):e104377. doi: 10.1371/journal.pone.0104377 (PMC4123918; doi:10.1371/journal.pone.0104377)

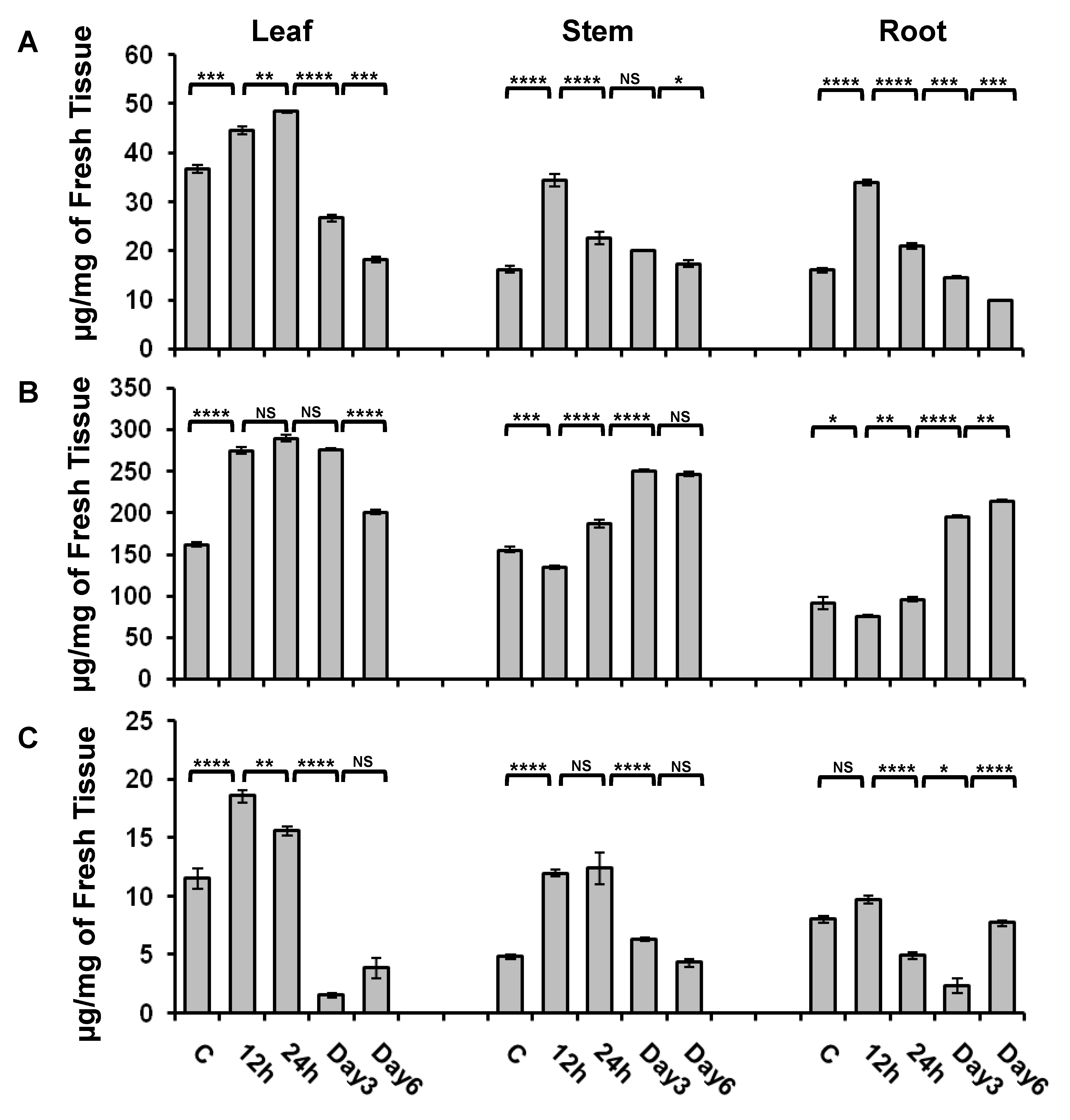

Supplement: Figure S1 — Protein, carbohydrate and lipid content of O. kilimandscharicum leaves following H. armigera feeding. Changes in the levels of A. total proteins B. total carbohydrates C. total lipids in leaves, stems and roots of tomato and O. kilimandscharicum at 12 h, 24 h, day 3, and day 6 post-infestation by H. armigera second-instar larvae. Two way ANOVA followed by Tukey's multiple comparisons test suggested significant difference between the data at. p<0.001 (indicated as ‘***’), p<0.01 (indicated as ‘**’), p<0.05 (indicated as ‘*’). One color represents data from respective day. NS represents group with non-significant difference in that particular day. Error bars represent Mean ± SD of 4 independent sets of tissue samples. (TIF) [file pone.0104377.s001.tif]

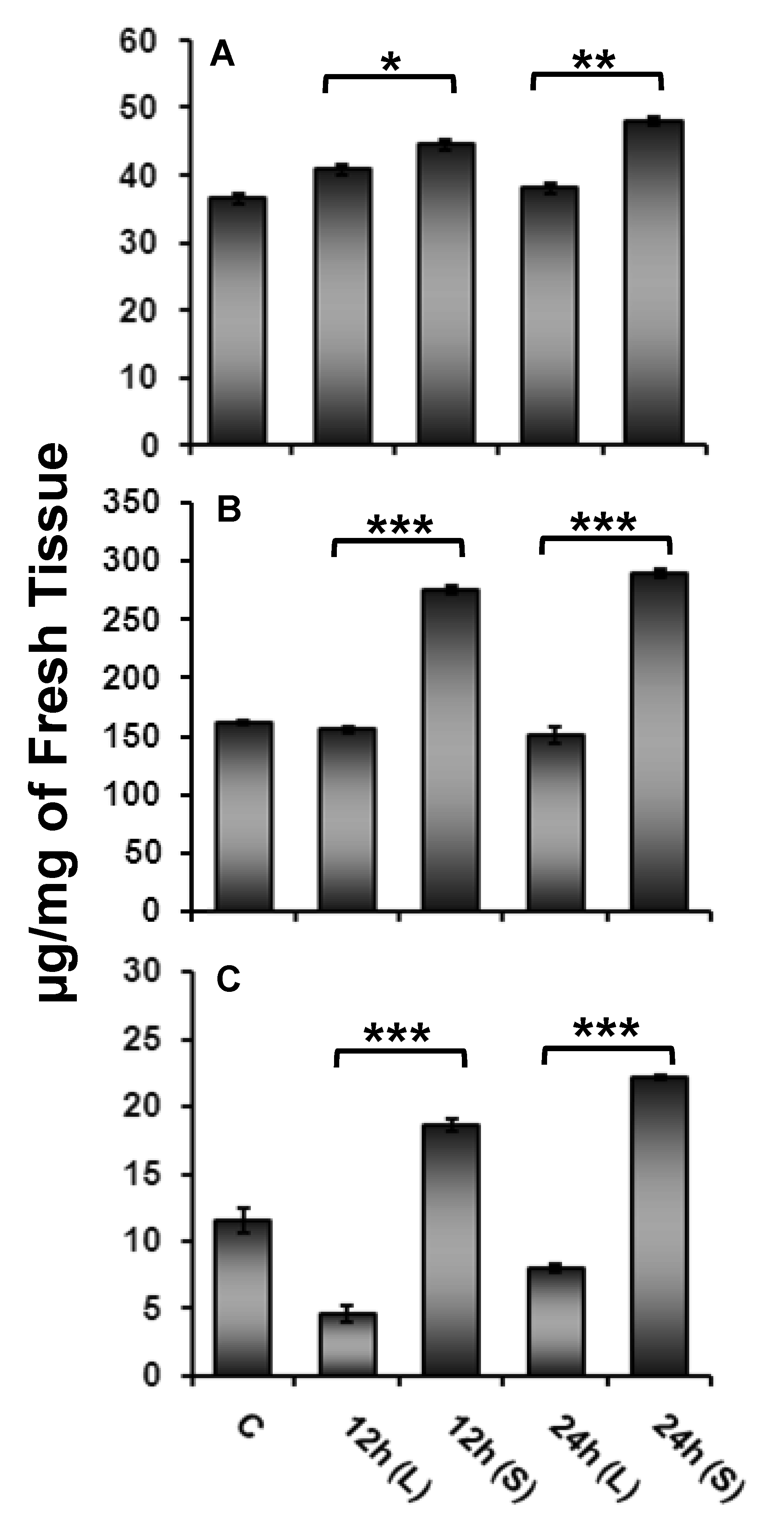

Supplement: Figure S2 — Protein, carbohydrate and lipid content of O. kilimandscharicum leaves following H. armigera feeding. Changes in the levels of A. total proteins B. total carbohydrates C. total lipids in local (L) versus systemic (S) leaf tissue in O. kilimandscharicum at 12 and 24 h post-infestation by H. armigera second-instar larvae. Unpaired t test suggested significant difference between the local and systemic tissue analysis data at. p<0.001 (indicated as ‘***’), p<0.05 (indicated as ‘*’). Error bars represent Mean ± SD of 4 independent sets of tissue samples. (TIF) [file pone.0104377.s002.tif]
